# Discontinuation rate and serious adverse events of chemoimmunotherapy as neoadjuvant treatment for triple-negative breast cancer: a systematic review and meta-analysis

**DOI:** 10.1016/j.esmoop.2023.102198

**Published:** 2023-12-14

**Authors:** A. Rizzo, F.M. Schipilliti, F. Di Costanzo, S. Acquafredda, G. Arpino, F. Puglisi, L. Del Mastro, F. Montemurro, M. De Laurentiis, M. Giuliano

**Affiliations:** 1 IRCCS Istituto Tumori “Giovanni Paolo II”, Bari; 2 Oncological Department, Sant’Andrea Hospital, University Sapienza in Rome, Rome; 3 Department of Clinical Medicine and Surgery, University of Naples Federico II, Naples; 4 Department of Medicine, University of Udine, Udine; 5 Department of Medical Oncology-CRO Aviano, National Cancer Institute, IRCCS, Aviano; 6 Department of Internal Medicine and Medical Specialties (DiMI), School of Medicine, University of Genova, Genova; 7 Medical Oncology Department, U.O. Clinica di Oncologia Medica, IRCCS Ospedale Policlinico San Martino, Genova; 8 Candiolo Cancer Institute, FPO-IRCCS, Candiolo; 9 Istituto Nazionale Tumori IRCCS “Fondazione Pascale”, Napoli, Italy

**Keywords:** breast cancer, chemoimmunotherapy, pembrolizumab, neoadjuvant, breast

## Abstract

**Background:**

The use of combination of chemotherapy with immune checkpoint inhibitors (ICIs) has shown efficacy in triple-negative breast cancer (TNBC), and chemoimmunotherapy has been introduced in clinical practice. However, limited data are available on the discontinuation rate and serious adverse events of these treatments, particularly in the neoadjuvant setting. Herein, we carried out a comprehensive systematic review and meta-analysis to assess discontinuation rate and serious adverse events of chemoimmunotherapy compared to chemotherapy alone in phase II and III neoadjuvant clinical trials in TNBC.

**Materials and methods:**

Following the Preferred Reporting Items for Systematic Reviews and Meta-Analyses (PRISMA) guidelines, EMBASE, Cochrane Library, and PubMed/Medline were searched for articles published from June 2008 to May 2023. The outcomes of interest were the discontinuation rate, serious adverse events, and grade 3-4 adverse events.

**Results:**

Four studies were included in the analysis. The pooled odds ratios (ORs) for discontinuation rate and serious adverse events were 1.26 [95% confidence interval (CI) 0.78-2.06] and 1.79 (95% CI 1.4-2.28), respectively, in patients receiving chemoimmunotherapy compared to chemotherapy alone as neoadjuvant treatment for TNBC. The chemoimmunotherapy group had a higher risk of grade 3-4 adverse events (OR 1.30, 95% CI 1.07-1.59). The analysis showed substantial heterogeneity, and the risk of discontinuation rate was heavily influenced by the KEYNOTE-522 trial.

**Conclusions:**

Our findings highlight the need for clinical trials specifically focused on safety, quality of life, and treatment adherence in TNBC patients receiving neoadjuvant treatment. Close monitoring of tolerability remains crucial in this clinical setting.

## Highlights

• We carried out a meta-analysis to assess discontinuation rate and serious adverse events of chemoimmunotherapy in TNBC.
• Our findings highlight the need for clinical trials specifically focused on safety, quality of life, and treatment adherence.
• Close monitoring of tolerability remains crucial in this clinical setting.

## Introduction

Breast cancer (BC) remains a leading cause of cancer-related death.^1^ BC is a highly heterogeneous disease, whose clinical strategy, prognosis, and response to systemic treatments widely vary among different subtypes.^2^ Triple-negative BC (TNBC) accounts for ∼15%-20% of all BCs, with this disease lacking or showing low levels of estrogen receptor, progesterone receptor, and absence of human epidermal growth factor receptor 2 overexpression and/or gene amplification.^3^^,^^4^ TNBC is also characterized by a high level of tumor-infiltrating lymphocytes (TILs), high programmed death-ligand 1 (PD-L1) expression, and high median tumor mutational burden, which have been associated, with different levels of evidence, with the response to immune checkpoint inhibitors (ICIs), such as anti-programmed cell death protein 1 (PD-1) and PD-L1 agents.^5^, ^6^, ^7^, ^8^ This strong biological rationale has led to the development of immunotherapy for TNBC patients, either as monotherapy or in combination with other anticancer agents, including systemic chemotherapy. As regards combinatorial strategies, immune-based combinations with ICIs and chemotherapy have emerged as a standard of care for patients with PD-L1-positive metastatic TNBC, and these results have supported the testing of these agents in early-stage BC as well.^9^ Neoadjuvant chemoimmunotherapy for TNBC patients has recently been explored, and several ICIs, including the PD-1 inhibitor pembrolizumab and the two PD-L1 inhibitors atezolizumab and durvalumab, have been tested.^10^, ^11^, ^12^, ^13^

In the KEYNOTE-522 trial, the addition of pembrolizumab to neoadjuvant chemotherapy (NACT) significantly increased the rate of pathological complete response (pCR) compared to chemotherapy alone. Since pCR rates, defined as the complete eradication of invasive cancer in the breast and axillary lymph nodes (ypT0/is ypN0) after neoadjuvant therapy, are strongly associated with higher overall survival and event-free survival in TNBC, the results of this trial are particularly important.^14^^,^^15^ However, these findings have not been consistently replicated in other clinical trials testing neoadjuvant ICIs.^16^ Although the safety profile of ICIs is generally acceptable, immunotherapy has a specific set of treatment-related toxicities resulting from the aberrant activation of the immune system, which may severely limit treatment adherence and sometimes lead to treatment discontinuation.^17^ Of note, few data are available regarding the impact of treatment-related adverse events and discontinuation rates in TNBC patients receiving neoadjuvant chemoimmunotherapy. Based on these premises, we carried out a systematic review and meta-analysis aiming to systematically assess discontinuation rate and serious adverse events in phase II and III clinical trials comparing chemoimmunotherapy versus chemotherapy alone as neoadjuvant treatment for TNBC patients.

## Materials and methods

### Search strategy

We conducted a search of phase II and III clinical trials published from 15 June 2008 to 18 March 2023 that evaluated neoadjuvant chemoimmunotherapy for TNBC patients with early-stage disease. The search was carried out by three authors using keywords in EMBASE, Cochrane Library, and PubMed/Medline. The keywords used included “immunotherapy” OR “nivolumab” OR “ipilimumab” OR “atezolizumab” OR “pembrolizumab” OR “durvalumab” OR “avelumab” OR “immune checkpoint inhibitors” AND “chemotherapy” OR “carboplatin” OR “epirubicin” OR “paclitaxel” OR “nab-paclitaxel” OR “anthracyclines” AND “neoadjuvant therapy” OR “neoadjuvant chemotherapy” OR “preoperative treatment” AND “breast cancer” OR “triple negative breast cancer” OR “early stage breast cancer” OR “TNBC”. Only English language articles published in peer-reviewed journals were included. Proceedings of the main international oncological meetings [such as European Society of Medical Oncology (ESMO), American Society of Clinical Oncology (ASCO), American Association for Cancer Research (AACR), European CanCer Organization (ECCO)] were also searched from 2008 onward for relevant trials and/or abstracts. The meta-analysis was conducted according to Preferred Reporting Items for Systematic Review and Meta-Analyses (PRISMA) guidelines (Supplementary Material, available at https://doi.org/10.1016/j.esmoop.2023.102198).

### Assessment of risk of bias in included studies

The methodological quality of the included trials was evaluated using Cochrane Collaboration tool, and risk of bias in the selected studies was assessed independently by three authors.

### Type of outcome measures

We evaluated the discontinuation rate and serious adverse events as primary outcome measures. Additionally, we analyzed grade 3-4 adverse events (Supplementary Material, available at https://doi.org/10.1016/j.esmoop.2023.102198). Three independent authors extracted data from the safety analysis of each trial. The data were obtained from the safety analysis of each study or supplementary materials. The research question was formulated using the PICO framework as follows:

- Population: non-metastatic TNBC patients undergoing neoadjuvant treatment;
- Intervention: chemotherapy in combination with immunotherapy;
- Comparison: chemotherapy alone;
- Outcome: discontinuation rate, serious adverse events, and grade 3-4 adverse events.

### Statistical design

All statistical analyses were carried out using R Studio. We calculated odds ratios (ORs) to analyze dichotomous variables, including the discontinuation rate, serious adverse events, and grade 3-4 adverse events. The ORs were combined using the Mantel–Haenszel method. Heterogeneity among the included trials was measured by chi-square test and *I*^2^ statistic, with substantial heterogeneity that was considered to exist when the *I*^2^ value was >50% or there was a *P* value <0.10 in the chi-square test. Quantitative data were analyzed using a fixed-effects model when *I*^2^ was <50%. Conversely, a random-effects model was used in the presence of substantial heterogeneity.^18^^,^^19^

## Results

### Selected studies

**Figure 1:**
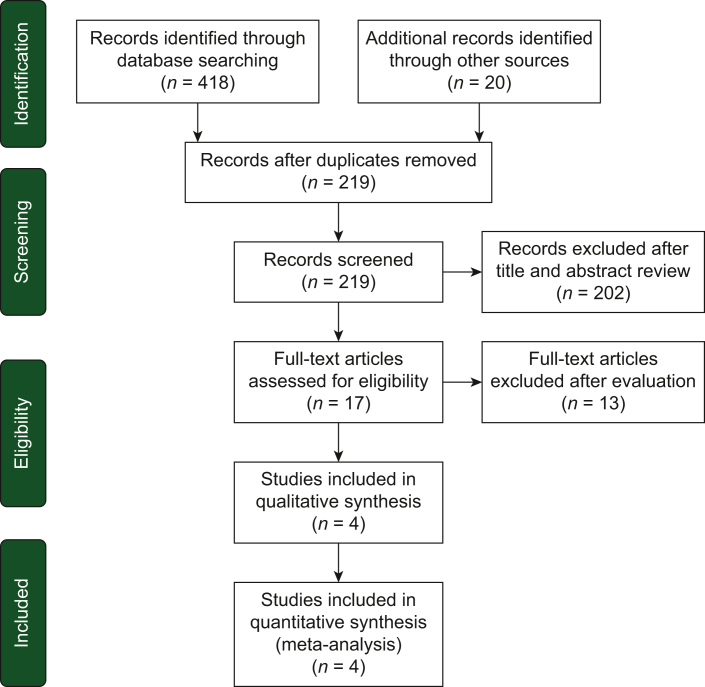
Diagram of all the trials included and excluded in the present meta-analysis.

A total of 438 potentially relevant reports were identified and independently evaluated by four authors.^10^, ^11^, ^12^, ^13^ After careful assessment, we excluded 434 records that were deemed non-pertinent, including editorials, case reports, ongoing studies/trials in progress, review articles, preclinical studies, retrospective studies, systematic reviews and meta-analyses, single-arm trials, non-randomized trials, and those lacking available data on the outcomes of interest. The selection process and a summary of the trials included in the meta-analysis are presented in Figure 1 and Table 1, respectively.^10^, ^11^, ^12^, ^13^

**Table 1:** Summary of all the included studies in the present meta-analysis

Trial	Study design	Number of patients	Type of ICI	Type of taxane	Treatment details	Primary endpoint	Results
KEYNOTE-522	Phase III	1174	Pembrolizumab	Paclitaxel	Paclitaxel and carboplatin plus pembrolizumab/placebo for 12 cycles every week followed by 4 cycles every 3 weeks of doxorubicin–cyclophosphamide or epirubicin–cyclophosphamide plus pembrolizumab/placebo Following surgery, adjuvant pembrolizumab for up to nine cycles	pCR at the time of definitive surgery EFS in the ITT population	64.8% of patients with pCR (95% CI 59.9% to 69.5%) in the pembrolizumab–chemotherapy group versus 51.2% in the placebo–chemotherapy group (95% CI 44.1% to 58.3%). Estimated treatment difference 13.6% points (95% CI 5.4% to 21.8%) 7.4% of patients in the pembrolizumab–chemotherapy group had disease progression/local distant recurrence versus 11.8% in the placebo–chemotherapy group
IMpassion031	Phase III	333	Atezolizumab	Nab-paclitaxel	Nab-paclitaxel plus atezolizumab or placebo every 2 weeks for 12 weeks followed by doxorubicin and cyclophosphamide plus atezolizumab or placebo every 2 weeks for 8 weeks	pCR at the time of definitive surgery pCR in ITT or PD-L1+ (PD-L1 IC ≥1%) patients	57.6% of patients with pCR (95% CI 49.7% to 65.2%) in the atezolizumab–chemotherapy group versus 41.1% in the placebo–chemotherapy group (95% CI 33.6% to 48.9%) Estimated treatment difference 16.5% points (95% CI 5.9% to 27.1%). In PD-L1+ patients (*n* = 152), pCR was reported in 68.8% (95% CI 57.3% to 78.9%) versus 49.3% (95% CI 37.6% to 61.1%) of patients
NeoTRIP	Phase III	280	Atezolizumab	Nab-paclitaxel	Carboplatin and nab-paclitaxel on days 1 and 8 plus atezolizumab or placebo on day 1 every 3 weeks for eight cycles before surgery. Following surgery, four cycles of an adjuvant anthracycline regimen	EFS	Follow-up for the EFS is ongoing The ITT analysis revealed that pCR rate after treatment with atezolizumab (48.6%) did not reach statistical significance compared to no atezolizumab [44.4%; odds ratio (OR) 1.18, 95% CI 0.74-1.89]
GeparNUEVO	Phase II	174	Durvalumab	Nab-paclitaxel	Durvalumab or placebo every 4 weeks added to nab-paclitaxel weekly for 12 weeks, followed by durvalumab or placebo every 4 weeks plus epirubicin/cyclophosphamide every 2 weeks followed by surgery. Durvalumab was not continued after surgery	pCR at the time of definitive surgery	pCR rate with durvalumab was 53.4% (95% CI 42.5% to 61.4%) versus placebo 44.2% (95% CI 33.5% to 55.3%), corresponding to OR 1.45 (95% CI 0.80-2.63)

CI, confidence interval; EFS, event-free survival; IC, immune cells; ICI, immune checkpoint inhibitor; ITT, intention-to-treat; pCR, pathological complete response.

### Risk of bias assessment

**Figure 2:**
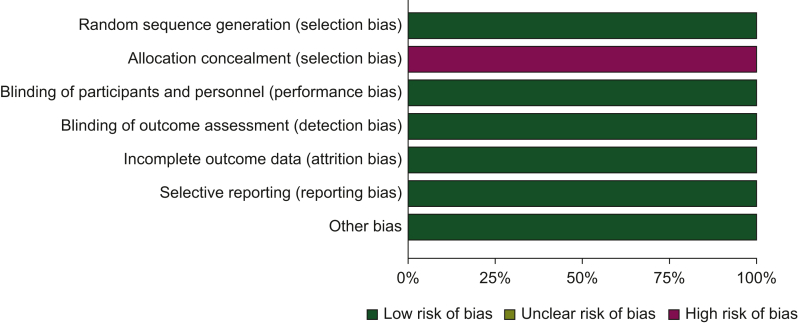
**Risk of bias graph.** The authors’ judgments about each risk of bias item are presented as percentages across all included studies.

The included studies were evaluated for their risk of bias, which was categorized as ‘low risk’, ‘high risk’, or ‘unclear risk’ across specified domains including selection, performance, attrition, and reporting bias. We compared the reported outcomes in the published papers with those specified in study protocols or trial registries. The results of this assessment are summarized in the risk of bias graph shown in Figure 2.

### Discontinuation rate

**Figure 3:**
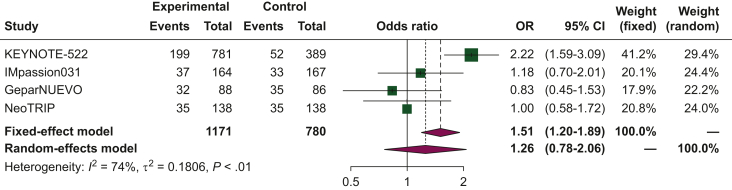
**Forest plot of comparison between chemoimmunotherapy versus chemotherapy alone as neoadjuvant treatment for triple-negative breast cancer (TNBC).** The outcome was the odds ratio (OR) of discontinuation rate. CI, confidence interval.

In patients with TNBC undergoing neoadjuvant treatment, the pooled ORs for the discontinuation rate was 1.26 [95% confidence interval (CI) 0.78-2.06] when comparing chemoimmunotherapy to chemotherapy alone (Figure 3). The analysis exhibited substantial heterogeneity (*I*^2^ of 74%), and thus, a random-effects model was used.

#### Serious adverse events

**Figure 4:**
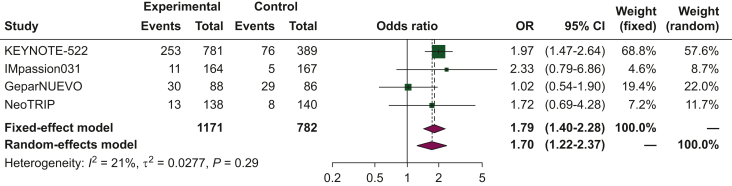
**Forest plot of comparison between chemoimmunotherapy versus chemotherapy alone as neoadjuvant treatment for triple-negative breast cancer (TNBC).** The outcome was the odds ratio (OR) of serious adverse events. CI, confidence interval.

The pooled OR for serious adverse events was 1.79 (95% CI 1.4-2.28) when comparing chemoimmunotherapy to chemotherapy alone as neoadjuvant treatment (Figure 4). Given the low heterogeneity observed in the analyses, a fixed-effects model was utilized.

##### Grade 3-4 adverse events

The pooled OR for grade 3-4 adverse events was 1.30 (95% CI 1.07-1.59) (Supplementary Figure S1, available at https://doi.org/10.1016/j.esmoop.2023.102198) when comparing chemoimmunotherapy to chemotherapy alone as neoadjuvant treatment. The analyses exhibited low heterogeneity, allowing the use of a fixed-effects model.

## Discussion

The emergence of ICIs has marked a significant breakthrough in the treatment of several hematological and solid tumors, including TNBC—which accounts for ∼15%-20% of all BCs and has traditionally been associated with a dismal prognosis.^20^ In fact, women with TNBC typically are diagnosed at younger ages and have a higher risk to present early recurrence following systemic treatments, and these patients exhibit the worst survival time among all BCs. A plethora of recent preclinical and clinical studies have reported that TNBC is more likely to respond to immunotherapy compared to other subtypes for several reasons.^21^^,^^22^ TNBC exhibits a higher abundance of TILs, which are associated with ICI response. Additionally, TNBC shows higher PD-L1 expression on immune and tumor cells, along with a higher number of tumor-specific neoantigens due to non-synonymous mutations.^23^ From a biological perspective, treatments such as chemotherapy and radiotherapy increase TIL proportions, providing a rationale for combining them with ICIs,^24^ with anti-PD-1 and anti-PD-L1 antibodies which enhance the ability of the immune system to effectively attack cancer. Following the results of the phase III IMpassion130 trial, the Food and Drug Administration approved the combination of atezolizumab plus nab-paclitaxel as a front-line treatment for PD-L1-positive, unresectable locally advanced or metastatic TNBC.^25^ The promising outcomes of chemoimmunotherapy as a first-line treatment in TNBC have motivated clinicians and researchers to explore its application in other settings, including neoadjuvant and adjuvant treatment. Neoadjuvant ICIs have the potential to elicit effective systemic immune responses and have been extensively investigated in other tumor types, such as melanoma and early-stage non-small-cell lung cancer. Previous clinical studies have shown that adding PD-L1 inhibitors to NACT may improve the pCR rate in TNBC,^26^ irrespective of PD-L1 status. However, conflicting results have emerged from trials such as IMpassion031 and NeoTRIP. Furthermore, while neoadjuvant chemoimmunotherapy is recommended for its efficacy, there are limited data on the safety profile of these combinations. It is well-known that the combination of ICIs and chemotherapy may increase the occurrence of adverse effects and toxicities.^27^^,^^28^

Herein, we carried out a comprehensive meta-analysis to explore the discontinuation rate and serious adverse events associated with chemoimmunotherapy as neoadjuvant treatment for BC. We included four eligible trials in this meta-analysis: KEYNOTE-522, IMpassion031, NeoTRIP, and GeparNUEVO, which utilized pembrolizumab, atezolizumab, and durvalumab as immunotherapy agents, respectively. Our findings revealed a higher risk of serious adverse events in patients receiving chemoimmunotherapy compared to those treated with chemotherapy alone. Additionally, the chemoimmunotherapy group exhibited a higher risk of grade 3-4 adverse events. These results raise concerns regarding the use of potentially curative treatments in early-stage BC. Based on our study, close clinical monitoring of adverse events should be emphasized when ICIs are combined with cytotoxic chemotherapy to optimize treatment and prevent discontinuation. The increased risk of grade 3-4 events also raises questions regarding health-related quality of life (HRQoL), which has been recognized as a crucial aspect in cancer management over the past few decades. In fact, HRQoL has become a major endpoint in most phase III clinical trials, including those investigating neoadjuvant treatments, where effective symptom control is essential. Regarding the analysis of the discontinuation rate (Figure 3), it is worth noting that the results are strongly conditioned by the KEYNOTE-522 trial. In this trial, the risk of discontinuation rate was more than doubled in TNBC patients receiving chemoimmunotherapy compared to chemotherapy alone (OR 2.22), and these results may explain the high heterogeneity associated with this analysis (*I*^2^ of 74%).

Of note, treatment-related adverse events led to discontinuation of the trial drug in 23.3% of patients in the pembrolizumab–chemotherapy group of KEYNOTE-522, compared to 12.3% of patients in the placebo–chemotherapy group. Similar rates of discontinuation were observed in other clinical trials, such as IMpassion031 (23%), NeoTRIP (25%), and GeparNUEVO (22.7%), as shown in Supplementary Table S1, available at https://doi.org/10.1016/j.esmoop.2023.102198. This issue is particularly important when considering the timing of discontinuation since early discontinuation may result in suboptimal clinical outcomes, especially in terms of tumor response to neoadjuvant treatment, which is crucial in a potentially curative setting.

The present meta-analysis holds its own strengths and limitations. The strengths of our analysis encompass the inclusion of only phase II and III randomized controlled trials and the substantial number of patients (1171 in the ICI arm and 782 in the chemotherapy arm). In addition, highly sensitive systematic searches have been carried out in order to include evidence from clinical trials of neoadjuvant chemoimmunotherapy versus chemotherapy alone for non-metastatic TNBC. Moreover, data quality was ensured through the involvement of four different authors in the study selection and data extraction processes.

Nonetheless, the results should be interpreted with caution due to some limitations. Firstly, despite employing random-effects modeling to address heterogeneity, the analysis still exhibited substantial heterogeneity, with *I*^2^ exceeding 50%. Secondly, individual patient data were not available, and thus, we relied on aggregate data extracted from clinical trial results. Lastly, the included phase II and III trials investigated different ICI strategies. In addition, three clinical trials, namely KEYNOTE-173, NEOPACT, and I-SPY2 trial (Supplementary Table S1, available at https://doi.org/10.1016/j.esmoop.2023.102198), were not included in the analysis due to their study design (single arm) or lack of data on discontinuation rate and serious adverse events (e.g. I-SPY2).^29^, ^30^, ^31^

Of note, the phase Ib KEYNOTE-173 study showed that adding pembrolizumab to NACT significantly improved the pCR rate in TNBC patients, with discontinuation of treatment occurring in 15% of patients due to adverse events or consent withdrawal.^29^ The NEOPACT aimed to assess the efficacy of an anthracycline-free neoadjuvant regimen consisting of pembrolizumab plus carboplatin plus docetaxel in TNBC, with discontinuation rates of any trial drug reported in 12% of patients and immune-related adverse events observed in 28% of cases (grade ≥3 = 6%) (Supplementary Table S1, available at https://doi.org/10.1016/j.esmoop.2023.102198).^31^

## Conclusions

According to our results, the addition of immunotherapy to NACT may significantly increase the risk of serious adverse events in TNBC patients. It is imperative that future investigations delve deeper into this matter, utilizing real-world evidence to gain a more comprehensive understanding within this context. At the same time, our findings underscore the importance of conducting dedicated clinical trials that specifically address safety issues in this particular patient population.
